# Classification of the colonic splenic flexure based on three-dimensional CT analysis

**DOI:** 10.1093/bjsopen/zraa040

**Published:** 2021-02-15

**Authors:** K Kawai, H Nozawa, K Hata, T Tanaka, T Nishikawa, K Sasaki, S Ishihara

**Affiliations:** Department of Surgical Oncology, Faculty of Medicine, University of Tokyo, Tokyo,Japan

## Abstract

**Background:**

Mobilization of the splenic flexure can be a challenging surgical step in colorectal surgery. This study aimed to classify the splenic flexure based on the three-dimensional (3D) coordinates of the splenic hilum and left renal hilum. This classification was used to compare splenic flexure mobilization during colorectal resection.

**Methods:**

CT images of patients with colorectal cancer treated between April 2018 and December 2019 were analysed retrospectively. 3D mutual positioning of the splenic flexure from the ligament of Treitz to the splenic hilum or the left renal hilum was used to classify patients into three groups using cluster analysis. The difference in the procedure time between groups was also analysed in a subset of patients undergoing laparoscopic colectomy with complete splenic flexure mobilization.

**Results:**

Of 515 patients reviewed, 319 with colorectal cancers were included in the study and categorized based on the 3D coordinates of the splenic hilum and left renal hilum as caudal (100 patients), cranial (118) and lateral (101) positions. Male sex (*P* < 0.001), older age (*P* = 0.004) and increased bodyweight (*P* = 0.043) were independent characteristics of the lateral group in multiple logistic regression analysis. Thirty-four patients underwent complete splenic flexure mobilization during the study period; this took significantly longer (mean 78.7 min) in the lateral group than in the caudal and cranial groups (41.8 and 43.2 min respectively; *P* = 0.006).

**Conclusion:**

Locating the splenic flexure using 3D coordinates could be helpful in predicting a longer duration for mobilization of the splenic flexure.

## Teaser

This study aimed to establish a classification to predict the difficulty of mobilizing the splenic flexure during colorectal surgery by analysing the three-dimensional coordinates of the splenic hilum and left renal hilum using cluster analysis. Three distinct groups were found: caudal, cranial and lateral locations.

## Resumen

**Antecedentes:**

La movilización del ángulo esplénico (*splenic flexure*, SF) puede ser un gesto quirúrgico difícil en cirugía colorrectal. Este estudio tuvo como objetivo clasificar la SF en función de las coordenadas 3D de los hilios esplénico y del riñón izquierdo. Se utilizó esta clasificación para analizar la movilización de SF durante las resecciones colorrectales.

**Métodos:**

Se evaluaron retrospectivamente las imágenes de tomografía computarizada de pacientes con cáncer colorrectal tratados entre abril de 2018 y diciembre de 2019. Mediante técnicas de agrupamiento, se clasificaron a los pacientes en tres grupos según el posicionamiento tridimensional (3D) de la SF entre el ligamento de Treitz, el hilio esplénico y el hilio renal izquierdo. En un subgrupo de pacientes en los que se realizó la movilización laparoscópica completa de la SF, se contabilizó la diferencia de tiempo en la realización del procedimiento.

**Resultados:**

De los 515 pacientes revisados, se incluyeron 319 pacientes con cánceres colorrectales y, según las coordenadas 3D del hilio esplénico y del hilio renal izquierdo, se categorizaron como en posición caudal (100 pacientes), craneal (118 pacientes) y lateral (101 pacientes). En un análisis de regresión logística múltiple, el sexo masculino (*P* < 0,001), la edad avanzada (*P* = 0,004) y el aumento de peso corporal (*P* = 0,043) fueron factores independientes en el grupo lateral. Durante el período de estudio se realizó la movilización completa de la SF en 34 pacientes, y esta movilización requirió tiempos significativamente más largos (79 min, en promedio) en el grupo lateral frente a los otros 2 grupos (42 y 43 min, respectivamente, *P* = 0,006).

**Conclusión:**

La ubicación de la SF utilizando coordenadas 3D podría ser útil para predecir una mayor duración en la movilización de SF.

## Introduction

Mobilization of the splenic flexure is sometimes necessary to obtain a tension-free anastomosis or to resect splenic flexure cancers. However, splenic flexure mobilization has been considered a challenging procedure with some technical difficulty^1^, and has been associated with a prolonged duration of surgery^2^^,^^3^, an increased incidence of splenic injury^4^^,^^5^ and increased blood loss^2^.

Although the use of laparoscopy has been reported to reduce iatrogenic splenic injury during mobilization of the splenic flexure^4^^,^^6^, this has not been confirmed by others who have reported a higher incidence of splenic flexure mobilization-related complications in laparoscopic surgery than in open procedures^7^. Thus, mobilizing the splenic flexure remains challenging, even in the era of laparoscopic colorectal resection. The procedure can be affected by several patient conditions, such as obesity, and the ability to predict the difficulty before surgery would be helpful.

The present study aimed to classify the splenic flexure by assessing three-dimensional (3D) coordinates in CT images based on the positional relationship of three retroperitoneal structures: the ligament of Treitz, splenic hilum and left renal hilum. This classification was used to compare the procedure time of splenic flexure mobilization during colorectal resection.

## Methods

Patients who had CT (using 1- or 3-mm slice thickness, enhanced and not enhanced) in the supine position for colorectal cancer, independently from the tumour location, between April 2018 and December 2019 in the Department of Surgical Oncology at University of Tokyo Hospital were analysed retrospectively. Exclusion criteria were: patients who had perforation, peritonitis, colonic ileus or invagination; a past history of colectomy, vertebral fusion or other upper abdominal operations such as gastrectomy and hepatectomy; and retroperitoneal anatomical variations, such as large renal cyst and abdominal aortic aneurysm.

For each patient, the following variables were reviewed and used for data analysis: age, sex, ethnicity, body height, bodyweight, BMI, the coordinate of the splenic or left renal hilum from the ligament of Treitz, and the distance from the ligament of Treitz to the splenic or left renal hilum.

The study was conducted with the approval of the ethics committee of the University of Tokyo Hospital (number 3252-(7)). Informed consent was obtained in the form of opt-out on the website.

### Assessment of three-dimensional coordinates

**Fig. 1:**
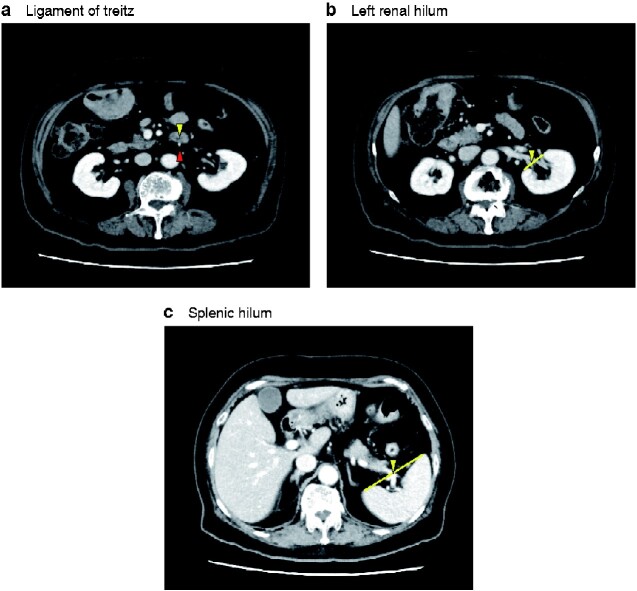
Definition of the ligament of Treitz, left renal hilum and splenic hilum **a** The ligament of Treitz (yellow triangle) was defined as the point of the duodenum that intersects ventral to the inferior mesenteric vein (red triangle). **b** The left renal hilum (yellow triangle) was defined as the midpoint of the renal hilum. **c** The splenic hilum (yellow triangle) was defined as the crossing point of the splenic vein.

The location of the splenic flexure is affected largely by bowel conditions, such as retention of air and faeces in the colon (*Fig.* *S1*). The splenic flexure lies above the hilum of the spleen, regardless of patient demographics^8^, and the descending colon runs just ventral or lateral to the left kidney. These points are retroperitoneal and are considered to be less affected by bowel conditions. Using the splenocolic ligament as a hallmark, the 3D positioning of the splenic hilum and left renal hilum was analysed^9^. The ligament of Treitz was also used as a reference point for the 3D evaluation of the hilum, because it is one of the retroperitoneal structures that can be identified most easily on laparoscopic view and is not affected by bowel conditions. Accordingly, these 3D coordinates were assessed for these three points in each patient: the ligament of Treitz, left renal hilum, and splenic hilum. The ligament of Treitz was defined as the point of the duodenum that intersects ventral to the inferior mesenteric vein (*Fig. 1a*). The left renal hilum was defined as the midpoint of the renal hilum (*Fig. 1b*), and splenic hilum was defined as the crossing point of the splenic vein and the line connecting lateral and internal edge of the spleen (*Fig. 1c*).

### Surgical mobilization of the splenic flexure

For this analysis, only those patients undergoing splenic flexure mobilization during left-sided colorectal resection using laparoscopy were included, because the procedure time for mobilization could not be assessed retrospectively in patients who had open surgery. Of note, complete splenic flexure mobilization is not performed routinely during surgery for sigmoid colonic or rectal cancer by surgeons in the authors’ department.

The splenic flexure mobilization procedure consisted of the following process: mobilization of the mesentery of the descending colon from the internal or caudal side, ligation and dissection of the inferior mesenteric vein at the lower edge of the pancreas, mobilization of the descending colon from the external side, dissection of the great omentum from the transverse colon, and dissection of the remaining splenocolic ligament. The videos were reviewed retrospectively, and the time spent for splenic flexure mobilization was assessed by a single surgeon blinded to the clinical information. The time for splenic flexure mobilization was defined as the time from the beginning of mobilization of the descending colon from the external side to the completion of splenic flexure mobilization, measured in minutes.

### Statistical analysis

Cluster analysis was performed using the k-means method, by scaling each variable independently of the other variables. Distances were scaled using an overall estimate of the standard deviation of each variable, without adjusting the distances based on the sizes of the clusters. Correlations between the clinical factors and the 3D coordinates or the time for splenic flexure mobilization were assessed using the χ^2^ test and ANOVA. The independence of each variable was evaluated using a multivariable logistic regression model, and variables with *P* < 0.050 in univariable analysis were used for multivariable analysis. The association between duration of surgery and the variables was also assessed using multiple linear regression analysis. All of these analyses were performed with the JMP^®^ Pro 14.0.0 software (SAS Institute, Cary, NC, USA), and *P* < 0.050 was considered statistically significant. 3D visualization was also performed using R 3.6.1 with the rgl package (R Foundation for Statistical Computing, Vienna, Austria; http://www.r-project.org/).

## Results

**Fig. 2:**
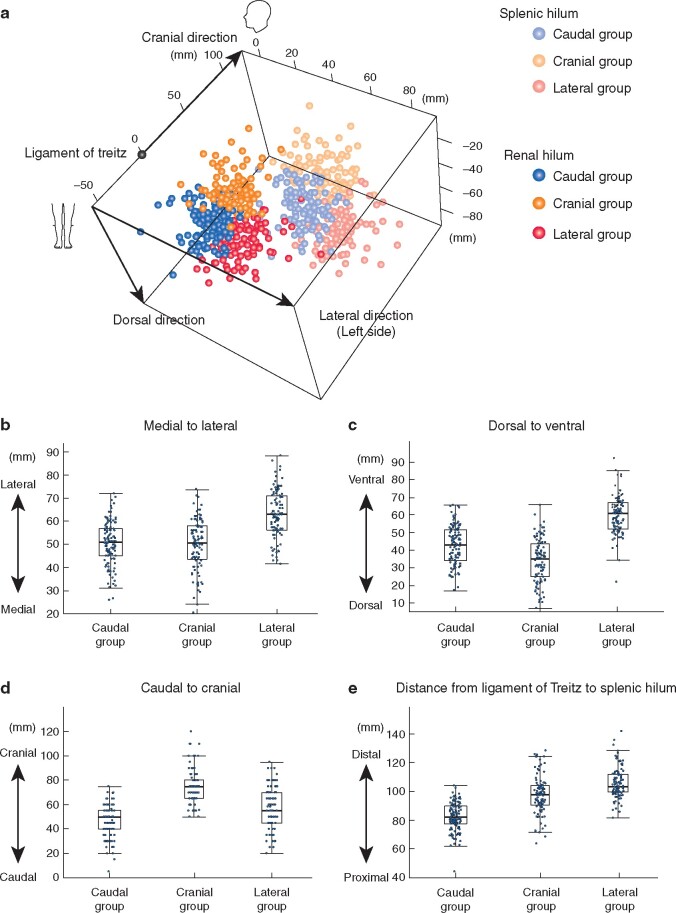
Stratification of the splenic hilum and left renal hilum using cluster analysis **a** Three-dimensional visualization of the location of the splenic and left renal hilum when the ligament of Treitz was defined as the reference point. **b–d** Distribution for the coordinates of the splenic hilum from the ligament of Treitz in each group: **b** distribution in medial-to-lateral direction; **c** dorsal-to-ventral direction; **d** caudal-to-cranial direction. **e** Distribution of distances from the ligament of Treitz to the splenic hilum. **b–e** *P* <0.001 (ANOVA).

Of 515 patients who underwent surgery for colorectal cancer during the study period, 319 met the inclusion criteria. All were Asian, with the exception of one Caucasian patient (*Table 1*). The splenic hilum was approximately 10 cm (median 94.4 mm) and the left renal hilum was approximately 5 cm (median 51.2 mm) from the ligament of Treitz. The 3D positions of the splenic hilum and left renal hilum were classified into three groups by using cluster analysis: caudal (100 patients), cranial (118) and lateral (101) (*Fig. 2*). Both the splenic and left renal hilum in the caudal group were located more medially and caudally, whereas the positions of the hilum in the cranial group were characterized by a more cranial and dorsal location; the lateral group showed a more lateral and ventral position. The distance from the ligament of Treitz to the splenic hilum was shorter in the caudal group (median 82.3 mm), followed by the cranial group (97.7 mm) and the lateral group (103.2 mm).

**Table 1:** Patient characteristics

	No. of patients* **(*n*=319)**
**Age (years)^†^**	67 (20–94)
**Sex ratio (M : F)**	163 : 156
**Ethnicity**	
Asian	318
Caucasian	1
**Body height (cm)^†^**	161 (140.5–192.0)
**Bodyweight (kg)^†^**	60 (31.6–111.5)
**BMI (kg/m^2^)^†^**	23.1 (14.3–42.9)
**Coordinate of splenic hilum from ligament of Treitz (cm)^†^**	
Lateral direction	54.6 (20.5–88.3)
Ventral direction	46.4 (6.8–92.2)
Cranial direction	60 (5–120)
**Distance from ligament of Treitz to splenic hilum (mm)^†^**	94.4 (44.2–141.8)
**Coordinate of left renal hilum from ligament of Treitz (mm)^†^**	
Lateral direction	24.6 (−8.2–65.6)
Ventral direction	38.9 (15.7–74.5)
Cranial direction	5 (−50–75)
**Distance from ligament of Treitz to left renal hilum (mm)^†^**	51.2 (23.3–89.9)

*Unless indicated otherwise;

^†^values are median (range).

Factors correlating with this classification were analysed (*Table 2*). Approximately one-third of the caudal group were women (68.0 per cent), whereas the proportion of men and women was nearly the same in the cranial group. In contrast, 77.2 per cent of the lateral group were men. Patients in the caudal group were the youngest, followed by those in the cranial group and then the lateral group. In terms of body height, patients in the caudal group had a mean height of 159.3 cm, whereas those in the lateral group had a mean height of 163.3 cm. On the other hand, bodyweight was lighter in the caudal group (mean 56.4 kg), followed by the cranial group (60.4 kg) and the lateral group (mean 68.2 kg).

**Table 2:** Association between clinical factors and location groups

	Caudal group (*n* = 100)	Cranial group (*n* = 118)	Lateral group (*n* = 101)	*P*
Age (years)*	62.7(14.6)	65.2(12.8)	69.1(11.6)	0.002
Sex ratio (M : F)	32 : 68	53 : 65	78 : 23	<0.001
Body height (cm)*	159.3(9.5)	160.4(9.6)	163.3(8.5)	0.008
Bodyweight (kg)*	56.4(12.3)	60.4(12.5)	68.2(14.1)	<0.001

*Values are mean(s.d.).

The multivariable logistic regression model found that male sex (*P* < 0.001), older age (*P* = 0.004) and increased bodyweight (*P* = 0.043) were independent characteristics of the lateral group, whereas body height was not (*P* = 0.405) (*Table 3*).

**Table 3:** Logistic regression analysis of factors associated with the lateral location

	Univariable *P*	Multivariable analysis	
		Odds ratio	*P*
Sex (F *versus* M)	<0.001	4.68 (2.16, 10.67)	<0.001
Age (<65 *versus* ≥65 years)	0.004	2.22 (1.28, 3.90)	0.004
Body height (<160 *versus* ≥160 cm)	<0.001	0.72 (0.32, 1.55)	0.405
Bodyweight (<60 *versus* ≥60 kg)	<0.001	1.95 (1.02, 3.76)	0.043

Values in parentheses are 95 per cent confidence intervals.

### Mobilization of the splenic flexure

**Fig. 3:**
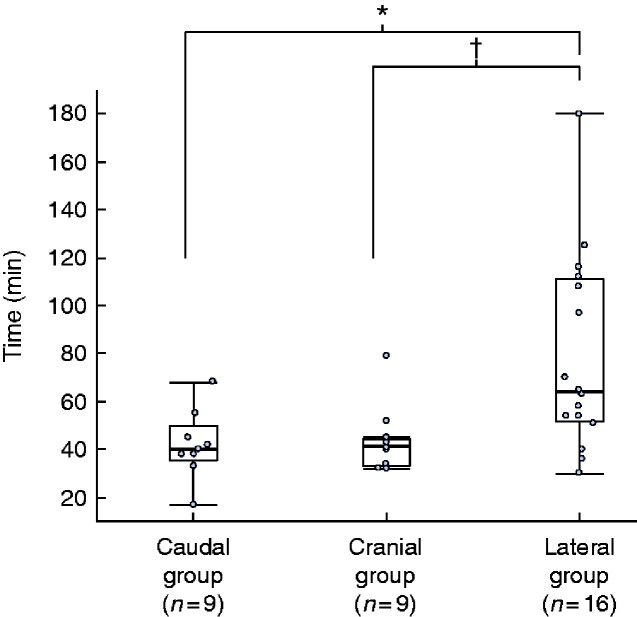
Difference in duration of mobilization of the splenic flexure between groups *P=0.016, ^†^*P*=0.021 (ANOVA).

Of 191 patients with left-sided colorectal cancer treated by laparoscopic resection during the study period, 34 had complete splenic flexure mobilization and were analysed (16 left colectomies, 6 partial resections of the transverse colon, 4 partial resections of the descending colon, 4 total colectomies, 3 sigmoidectomies, and 1 high anterior resection). There were no mobilization-related complications such as intraoperative splenic injury. The mean(s.d.) time for splenic flexure mobilization was 41.8(14.1), 43.2(14.3) and 78.7(40.6) min in the caudal, cranial and lateral group respectively, indicating a significantly longer duration in the lateral group (*P* = 0.006) (*Fig. 3*). Multiple linear regression analysis demonstrated that the lateral location was the only factor that showed an independent correlation with longer time for splenic flexure mobilization (*P* = 0.007) (*Table 4*).

**Table 4:** Multiple linear regression analysis of time spent mobilizing the splenic flexure

	Univariable *P*	Multivariable analysis	
		Δ time (min)	*P*
Sex (F *versus* M)	0.051	14.3	0.309
Age (years)	0.661		
Body height (cm)	0.569		
Body weight (kg)	0.015	0.56	0.148
Other groups *versus* lateral group	0.004	35.8	0.007

## Discussion

In recent years, cluster analysis with the k-means method has been used frequently for analyses of 3D measurement or body image^10^^,^^11^. Using this method, three distinct groups showing different mutual positional relationships between the ligament of Treitz and the splenic or left renal hilum were identified. Locations in the caudal group were caudal or medial, those in the cranial group were cranial, medial or dorsal, and those in the lateral group were characterized as lateral or ventral.

Body height showed only a slight influence on these groups, whereas sex had a strong correlation. A previous study^12^ reported that the splenic flexure was located more cranially and more laterally in men than in women in an analysis of 100 CT images. Although the present series showed also that the individuals in the lateral group were predominantly men, the cranial group had a distinct location pattern of cranial, dorsal or medial, with nearly the same proportion in male and female patients.

This classification can be affected by spleen size. Because the splenic hilum of the large spleen tends to be located medially and ventrally, individuals with a large body and small spleen tended to be classified in the lateral group, those with a large body and large spleen tended to be in the cranial group, and those with a small body tended to be in the caudal group. Several studies^13–15^ have demonstrated that the normal spleen size is affected by body size and age; for example, men and older, taller and heavier individuals tend to have longer and larger spleens. Further studies to elucidate the correlation between the classification proposed here and spleen size could be helpful. In the present analysis, the lateral location, which is considered to be the most difficult for splenic flexure mobilization, was found to correlate with the patient being an older obese man. Masoomi and colleagues^5^ investigated 975 825 patients who underwent colorectal resection and reported the splenic injury rate to be 0.96 per cent, with male sex as an independent risk factor for splenic injury. As the lateral location was found to be an independent predictive factor for long procedure time for splenic flexure mobilization in the present study, this points to clinical utility for the suggested classification.

This study had several limitations. First, because of the retrospective nature of case series, the surgical cohort included a wide variety of procedures, such as left hemicolectomy, sigmoid colectomy and total colectomy, and was performed by a number of surgeons with surgical experiences ranging from 10 to more than 20 years. Accordingly, a prospective study to validate the applicability of this classification is advocated. Second, almost of patients in the study were Asian, and the replicability of these findings remains unclear because spleen size can be different between people of different ethnicities^16–18^. Consequently, there could be a racial variation in the difficulty of splenic flexure mobilization.

## Funding

Japan Society for the Promotion of Science, 16K07143, 16K07161, 17K10620, 17K10621, 17K10623, 18K07194

## Supplementary Material

zraa040_Supplementary_Data
