# Supplementary material for: Classification of the colonic splenic flexure based on three-dimensional CT analysis
Source: BJS Open. 2021 Feb 15;5(1):zraa040. doi: 10.1093/bjsopen/zraa040 (PMC8271130; doi:10.1093/bjsopen/zraa040)

**zraa040_Supplementary_Material**

**Fig. S1 Spatial vectors from the ligament of Treitz to the splenic hilum or top of the splenic flexure in different bowel conditions**

This patient underwent computed tomography twice, without any bowel preparation (a) and after bowel preparation and air inflation from the anus (b). The vector from the ligament of Treitz to the splenic hilum shows a small difference, within 2 cm in every direction, between the bowel conditions. Meanwhile, the vector from the ligament of Treitz to the top of the splenic flexure shows more than 3 cm differences in the lateral and cranial direction between the two series.


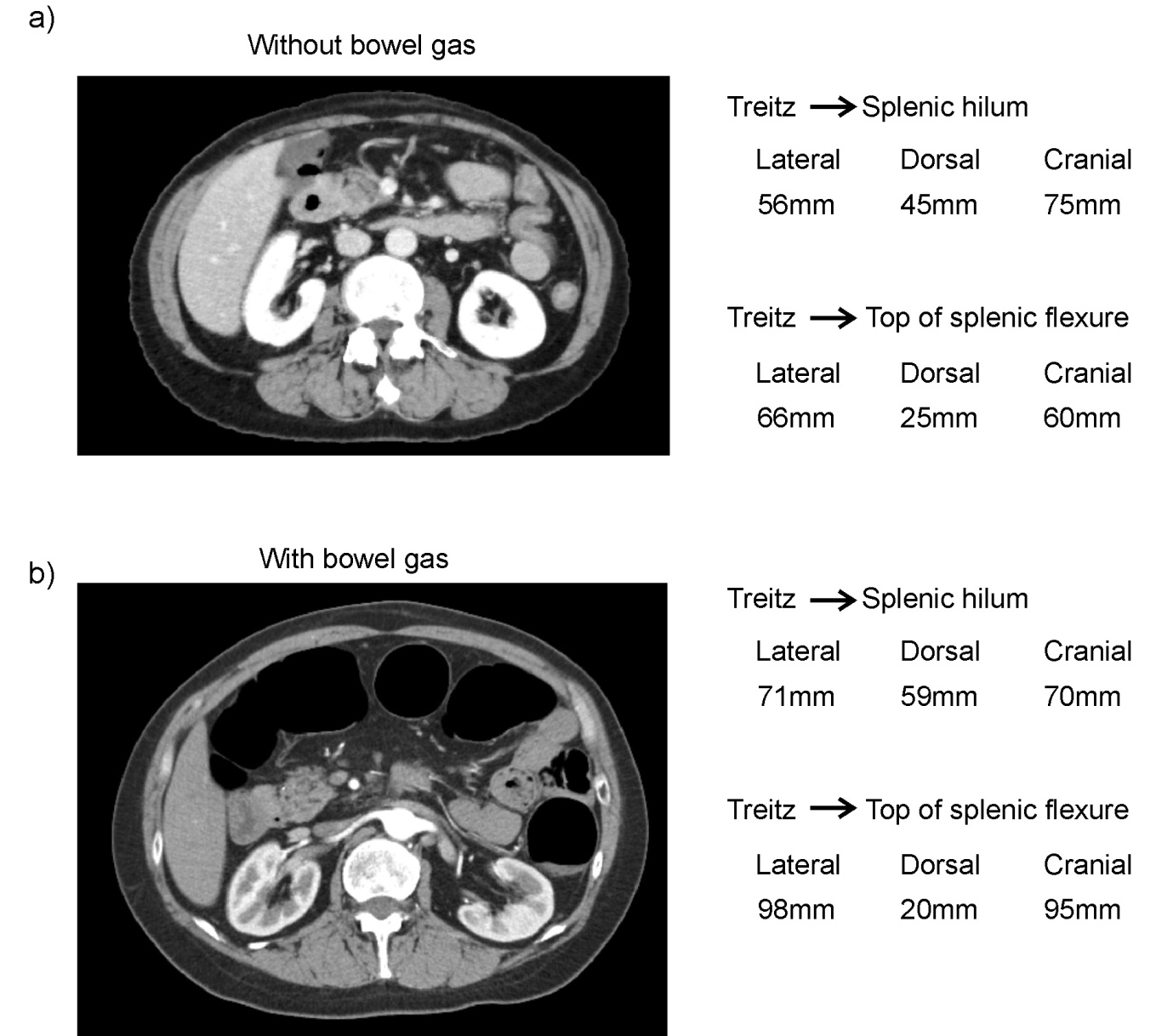


**Fig. S2 Representative case presentation of the cranial, caudal and lateral groups**

The red dots represent the ligament of Treitz, while the yellow arrows show the spatial vectors from the ligament of Treitz to splenic hilum.


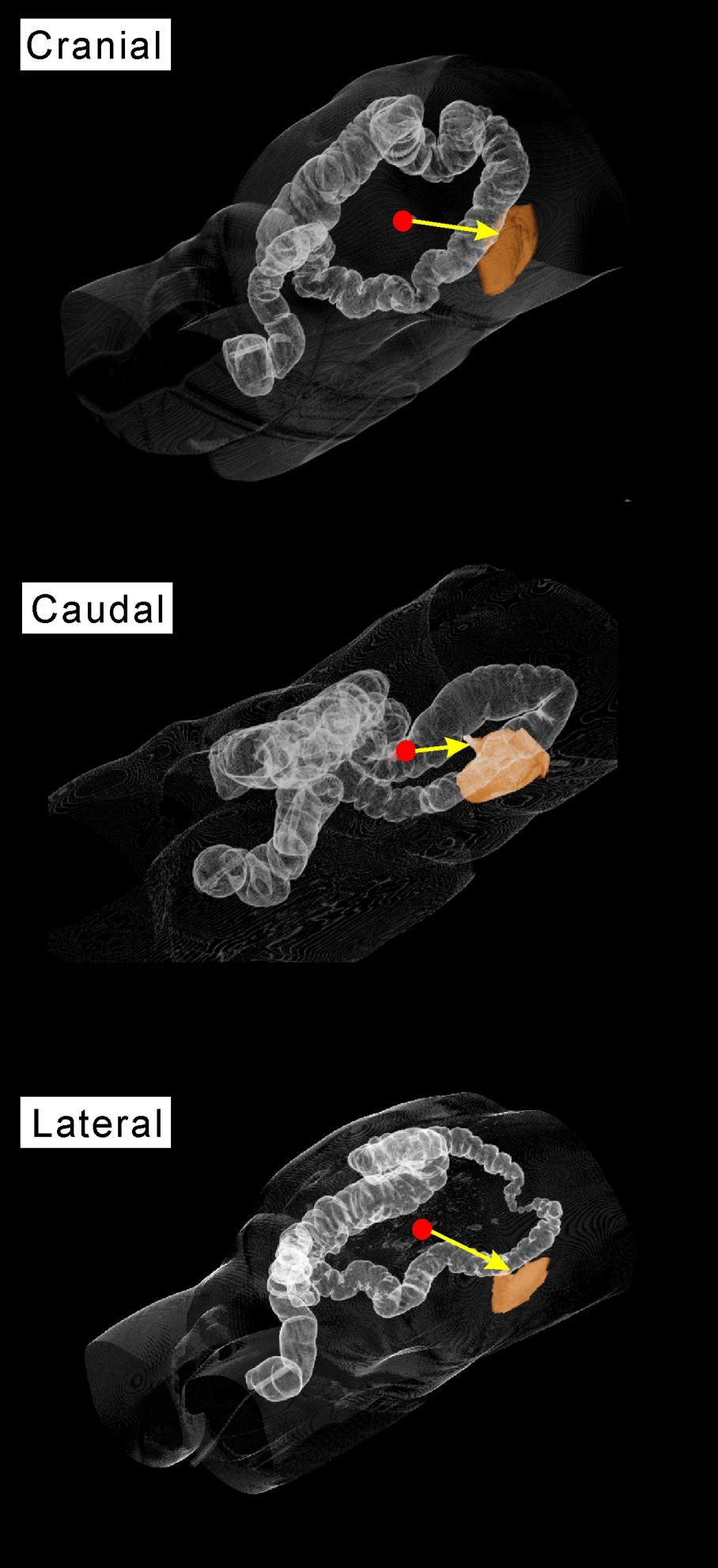

Supplement: zraa040_Supplementary_Data [file zraa040_supplementary_data.zip › zraa040_Supplementary_Material.docx]
