# Supplementary figures and images for: Classification of the colonic splenic flexure based on three-dimensional CT analysis
Source: BJS Open. 2021 Feb 15;5(1):zraa040. doi: 10.1093/bjsopen/zraa040 (PMC8271130; doi:10.1093/bjsopen/zraa040)

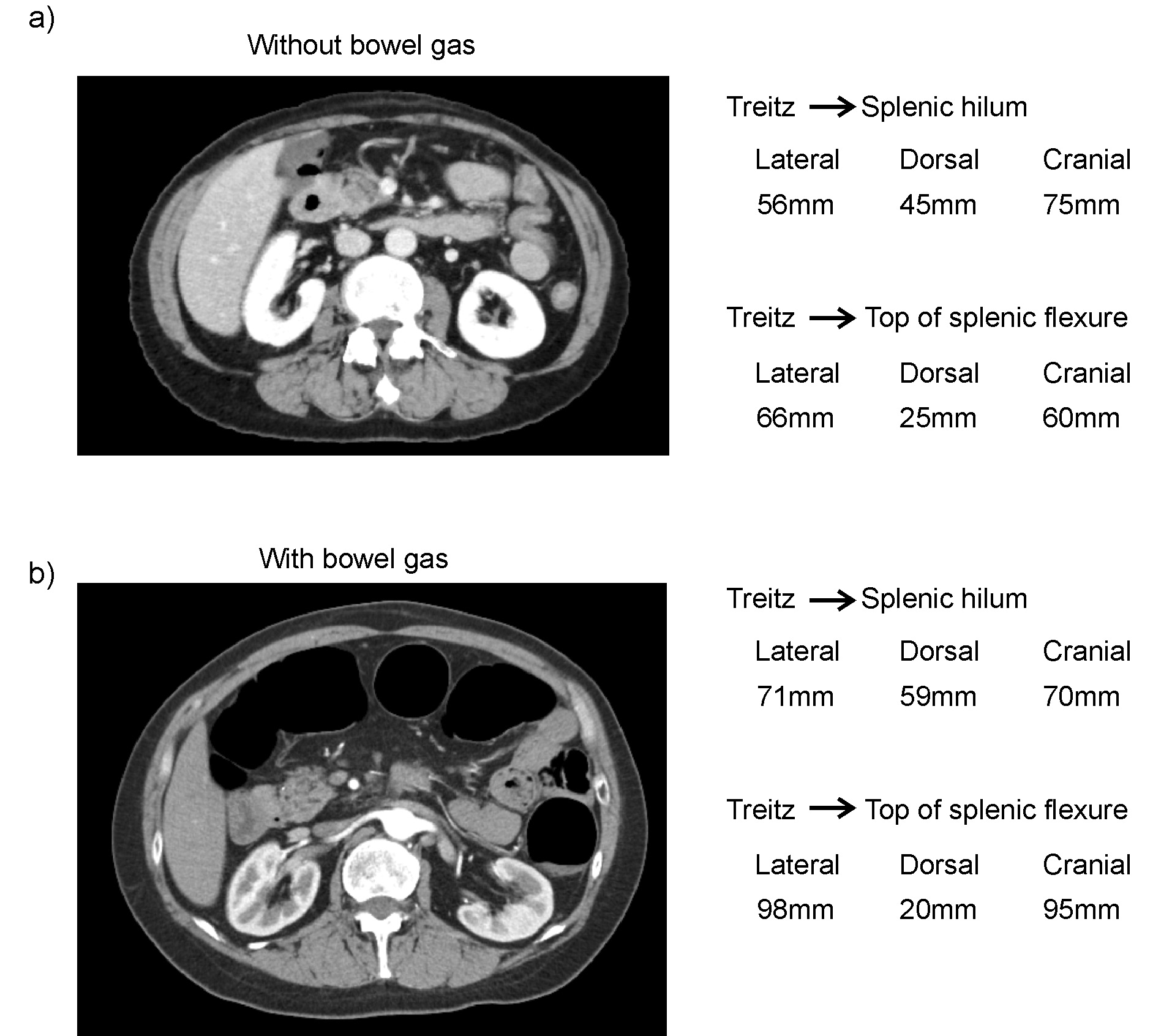

Supplement: zraa040_Supplementary_Data [file zraa040_supplementary_data.zip › Sup_Figure_1.jpg]

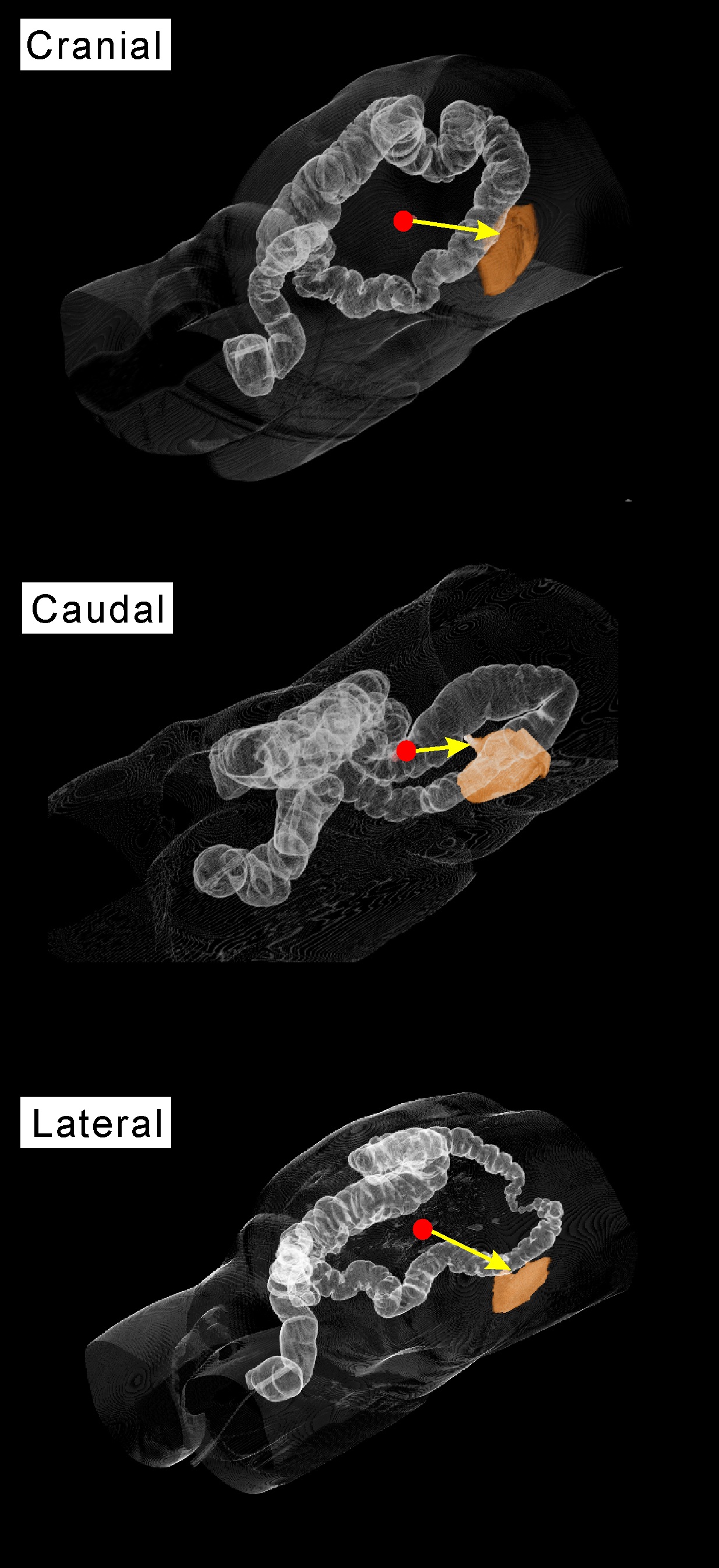

Supplement: zraa040_Supplementary_Data [file zraa040_supplementary_data.zip › Sup_Figure_2.jpg]
